# A Comparison of Mobile Social Media Promotion and Volunteer-Driven Strategies for Community Organizations Recruiting Men Who Have Sex with Men for HIV Testing in Zhejiang Province, China: Cross-Sectional Study Based on a Large-Scale Survey

**DOI:** 10.2196/66702

**Published:** 2025-02-13

**Authors:** Lin He, Shaoqiang Jiang, Tingting Jiang, Wanjun Chen, Jinlei Zheng, Hui Wang, Chengliang Chai

**Affiliations:** 1 Zhejiang Provincial Center for Disease Control and Prevention Hangzhou China; 2 Coastal Service Center Hangzhou China

**Keywords:** men who have sex with men, MSM, internet, recruit, HIV testing, community organization, strategy, China, mobile phone

## Abstract

**Background:**

China has recently implemented a strategy to promote and facilitate community organization involvement in HIV prevention among men who have sex with men (MSM). Although community-based strategies have been shown to increase HIV testing uptake, the relative effectiveness of mobile social media promotion compared with volunteer-driven recruitment remains underexplored. Limited research has investigated how these strategies differentially affect MSM who have not undergone previous HIV testing.

**Objective:**

This study aimed to compare the differences between a mobile social media promotion strategy and a volunteer-driven strategy for community organizations to recruit MSM for HIV testing.

**Methods:**

A cross-sectional study was conducted from July to December 2023 among MSM in Zhejiang Province, China. Participants aged 16 years with an HIV-negative or unknown status were recruited either through a mobile social media promotion strategy or through a volunteer-driven strategy by a community organization. They completed a questionnaire that collected information on demographics, sexual behavior, and HIV testing history. All participants were tested for HIV after completing the questionnaire. A multivariate logistic regression model was used to identify factors associated with recruitment through mobile social media promotion.

**Results:**

The study included 4600 MSM, of whom 3035 (66%) were recruited through the mobile social media strategy. Overall, 1.4% (66/4600) of participants tested positive for HIV, and 18.8% (865/4600) underwent HIV testing for the first time. Recruitment via the mobile social media promotion strategy was significantly associated with several factors: having only gay sexual partners (adjusted OR [aOR] 1.23, 95% CI 1.05-1.45), having more than 2 sexual partners in the past 3 months (aOR 1.74, 95% CI 1.42-2.11), frequently using rush poppers during sex (aOR 1.39, 95% CI 1.14-1.99), having a history of sexually transmitted infections (aOR 1.56, 95% CI 1.02-2.39), having awareness of pre-exposure prophylaxis (aOR 1.42, 95% CI 1.19-1.71), having awareness of postexposure prophylaxis (PEP; aOR 1.49, 95% CI 1.24-1.79), using mail-in HIV self-testing kits (aOR 2.02, 95% CI 1.77-2.31), testing HIV-positive (aOR 2.02, 95% CI 1.10-3.72), and first-time HIV testing (aOR 1.28, 95% CI 1.09-1.52).

**Conclusions:**

Community organizations play a critical role in expanding HIV testing and identifying undiagnosed individuals infected with HIV. Compared to the volunteer-driven outreach, mobile social media promotion strategies had a higher proportion of first-time testers and a higher rate of HIV positivity. We recommend prioritizing mobile social media strategies in regions with limited LGBTQ+ organizations or HIV health services to increase HIV testing coverage and interventions among MSM.

## Introduction

Men who have sex with men (MSM) represent a significant proportion of individuals living with HIV in China. According to recent estimates, the national prevalence of HIV among MSM was 5.7% in 2018 [1] and rose to 7% in 2022 [2]. Moreover, the percentage of newly diagnosed HIV infections attributed to homosexual transmission increased from 2.5% in 2006 to 25.6% in 2022 [2]. To achieve the United Nations Program on HIV/AIDS 95-95-95 targets (95% diagnosis, 95% treatment, and 95% viral suppression) by 2030, a renewed focus on MSM is essential [3]. While China is on track to meet the second and third 95% targets (treatment and viral suppression) [4], achieving the goal of diagnosing 95% of individuals infected with HIV by 2030 remains a significant challenge. Our previous study demonstrated that 12.9% of MSM will have never undergone HIV testing by 2023 [5]. It is, therefore, crucial to identify MSM who have not been tested, implement targeted HIV education and testing, and expand testing efforts to facilitate early diagnosis. This approach is pivotal for China to meet its 2030 diagnostic target.

Historically, MSM in China has faced significant barriers to HIV awareness, intervention, and counseling services. Traditional social norms and cultural values make it difficult for MSM to disclose their sexual orientation [6], leading many to conceal their identity. This hidden population poses a challenge for the current HIV service network, which is managed by the Centers for Disease Control and Prevention (CDC) and various medical institutions [7,8]. In recent years, China has implemented a series of comprehensive measures aimed at preventing and controlling HIV infections among MSM. One of the primary strategies is to promote and facilitate community organizational involvement in HIV prevention and control initiatives [2]. These encompass the implementation of public education initiatives, disease prevention and intervention strategies, and the provision of testing and counseling services. The community organization is composed of MSM volunteers, which allows for forming trusting relationships and facilitates contact with the MSM population, thus bringing attention to this previously hidden demographic [9,10]. A previous study revealed that 567 MSM community-based organizations in China participated in initiatives funded by the AIDS fund for nongovernmental organizations, facilitating HIV testing for over 250,000 people by 2022 [11]. Of these, 7963 were newly diagnosed, representing 28.9% of all MSM HIV cases nationwide. The involvement of community organizations has increased the reach of MSM communities, facilitating greater access to HIV prevention services and the identification of new infections [12].

HIV testing is a highly effective intervention strategy [13]. The implementation of HIV testing can facilitate early diagnosis, prompt treatment, and subsequent transmission reduction [14]. Studies have shown that MSM who have been tested for HIV are less likely to engage in high-risk sexual behaviors compared to those who have never been tested [15]. Consequently, a significant proportion of funding allocated to community organizations in China is directed toward HIV testing services [9]. Community organizations primarily use 3 methods to recruit MSM for HIV testing. The initial approach was a volunteer-driven strategy that involved active recruitment. Community-organized volunteers and peer educators were recruited from venues frequented by MSM, including parks, bars, and bathhouses, for in-person HIV testing and interventions. In addition, MSM who have not been tested for HIV for over 6 months are also mobilized for testing by these volunteers. Second, a mobile social media promotion or recruitment strategy is also used. Information on HIV promotion, prevention, and testing is disseminated through MSM websites, dating apps, and communication groups, which are then used to recruit MSM to offline service outlets. Alternatively, HIV self-testing kits may be ordered mobile and mailed home. Third, a regular testing strategy is implemented. Some MSM who were initially recruited through the aforementioned strategies continue to test regularly after receiving HIV education without the need for further mobilization by community organizations.

With the rapid growth of China’s internet economy and the widespread use of smartphones, mobile dating apps for MSM have surged in popularity [10]. Many MSM now use the internet and dating apps to find sexual partners. This shift has changed HIV testing recruitment from in-person volunteer outreach to internet-based strategies. Studies have shown that the internet is an effective tool for recruiting MSM, especially those who have never been tested, thus expanding the reach of HIV testing and reducing the number of undiagnosed infections [16,17]. Zhejiang Province, an economically developed and thriving internet economy in China, relies heavily on community organizations to implement HIV prevention and provide testing for MSM. In 2023, approximately 66,000 HIV tests have been conducted among MSM in the province, accounting for about 10% of the national total [4]. Notably, over 90% of these tests are performed by community organizations. To improve testing coverage among MSM, these organizations must use a dual recruitment strategy that uses both mobile social promotion and volunteer-driven initiatives. This approach is essential for identifying and engaging MSM who have not yet been tested for HIV. This study, based on the findings of a large-scale survey, aimed to analyze the effectiveness of 2 recruitment strategies, which are mobile social media promotion and volunteer-driven outreach. The results of this analysis will provide a foundation for promoting HIV testing within communities and contribute to recruiting MSM who have never been tested for HIV in China.

## Methods

### Study Design

A cross-sectional study based on a large-scale survey was conducted among MSM between July and December 2023.

### Study Participants

MSM were eligible to participate if they met certain criteria, which include being (1) 16 years or older, (2) reported having sex with men in the past year, (3) were HIV-negative or had an unknown HIV status, (4) received HIV testing as a direct result of mobile social media promotion or volunteer-driven recruitment, and (5) resided in Zhejiang Province, China.

### Participant Recruitment and Data Collection

Sunshine Coast Public Welfare is a social service agency that employs 13 full-time and 22 part-time social workers, with over 400 registered volunteers. The organization established a digital HIV prevention service using location-based services. MSM in Zhejiang Province are invited to visit the official “Sunshine Test” account on WeChat, a popular Chinese communication platform, to request free HIV testing and prevention counseling. MSM can choose between mailed self-testing kits or visiting offline services for HIV testing. Full-time and part-time social workers are responsible for regularly recruiting a specified number of MSM for HIV testing. Meanwhile, registered volunteers assist in promoting HIV testing within the MSM community periodically.

### Questionnaires

All individuals who engaged with Sunshine Test were required to complete a routine surveillance questionnaire consisting of 20 questions. These questions covered demographic information such as age, marital status, and education level, as well as sexual behavior including sexual roles (receptive, insertive, or both), number of sexual partners, use of rush poppers, sexual history, awareness of pre-exposure prophylaxis (PrEP) and PEP (postexposure prophylaxis), and HIV testing history.

Recruitment through direct engagement by social workers or registered volunteers was classified as “volunteer-driven recruitment.” Conversely, if MSM were recruited after encountering HIV prevention information posted by the community organization on internet platforms (such as dating apps, communication groups, or websites) and subsequently applied for testing, this was categorized as “mobile social media promotion recruitment.” If MSM had undergone an HIV test at the organization of their own volition, without being prompted by volunteers or mobile social media promotion, their case was classified as a “regular test.” During the study, the participating MSM underwent repeated HIV testing on more than 2 occasions, but only the first record was included in the analysis. Details of the Sunshine Coast Public Welfare Program have been described in previous literature [10].

### Statistical Analysis

Descriptive analyses were performed, with categorical variables presented as frequencies and proportions and continuous variables presented as median with IQR or mean with SD. The chi-square test (Χ^2^) was used to assess significant differences in demographic characteristics between MSM recruited through mobile social media promotion and those recruited through volunteer-driven recruitment. Factors associated with differences in the sociodemographic characteristics of mobile social media promotion recruitment were included in multivariate logistic regression models, and adjusted odds ratios (aORs) with 95% CIs were calculated. Statistical significance was set at *P*<.05, with β=.1. All statistical analyses were conducted using SPSS (version 19.0; IBM Corp).

### Ethical Considerations

This study was approved by the Zhejiang Provincial Centre for Disease Control and Prevention (approval number 2022-011-01). Written informed consent was obtained from all participants before survey completion, and participants had the ability to drop out anytime during the survey. All data were anonymous; participants received free HIV testing, health counseling, and no compensation; and all study procedures were conducted in accordance with approved guidelines and regulations.

## Results

**Figure 1:**
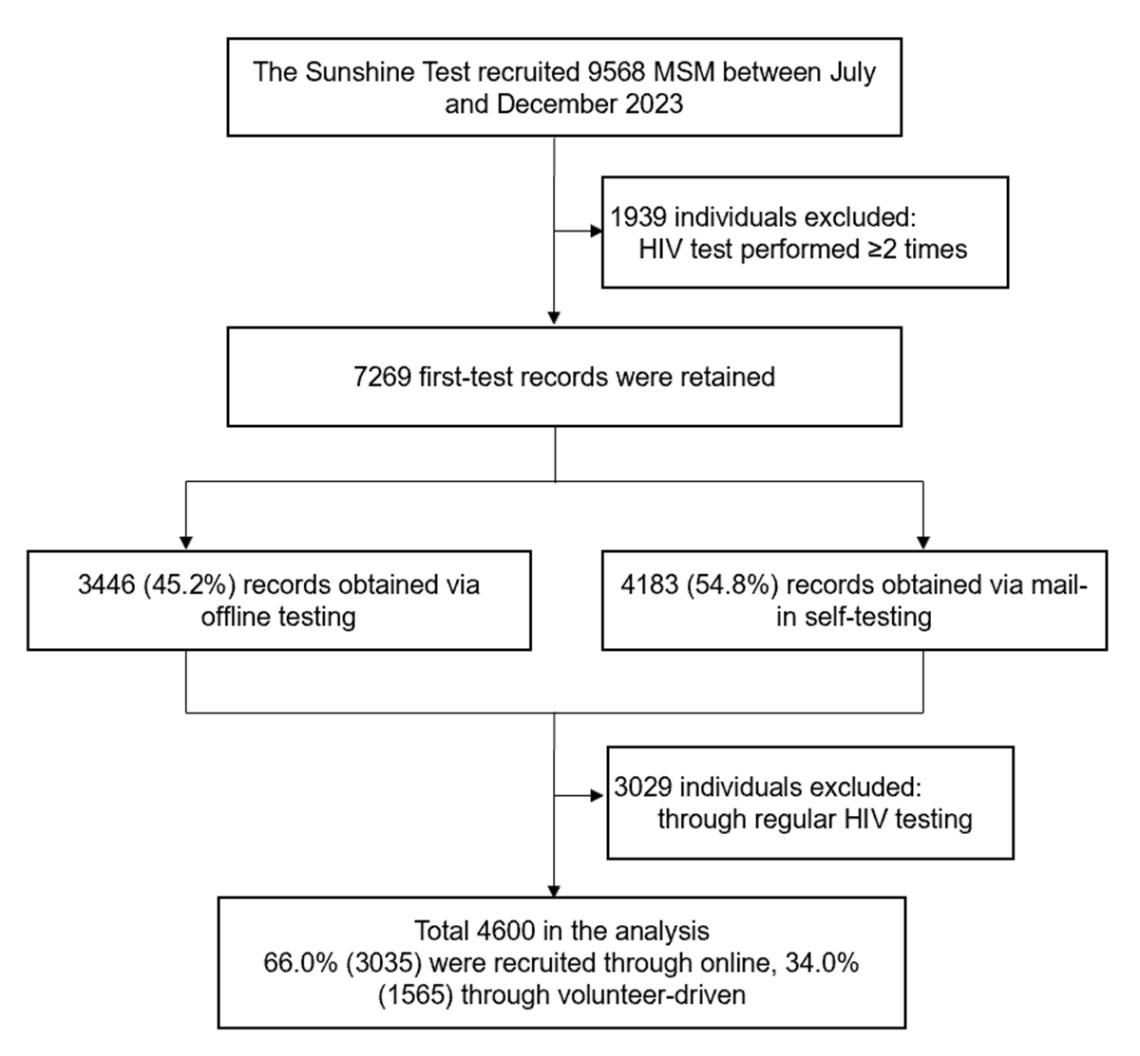
Study design. MSM: men who have sex with men.

A total of 4600 MSM were enrolled in the study (Figure 1), of whom 3035 (66%) were recruited via mobile social media promotion and 1565 (34%) through volunteer-driven recruitment. The mean age of the participants was 30.0 (SD 9) years. Overall, 78.4% (3606/4600) were single, and 74.5% (3425/4600) had a college degree or higher education. Most participants (3455/4600, 75.1%) reported having male sexual partners. Over the past 3 months, 30.8% (1419/4600) reported having more than 2 sexual partners, and 30.7% (1410/4600) reported using rush poppers during sexual activity. In addition, 43.6% (2007/4600) were aware of their sexual partner’s HIV status, 2.7% (126/4600) had a history of sexually transmitted infections (STIs), 47.2% (2169/4600) had used a mailed self-testing kit for HIV testing, 1.4% (66/4600) tested positive for HIV, and 18.8% (865/4600) were undergoing HIV testing for the first time (Table 1).

Overall, 66% (3035/4600) of MSM recruited through mobile social media promotion were aged 20-29 years (1842/2719, 67.7%), single (2404/3606, 66.7%), had a master’s degree or higher education (283/424, 66.7%), reported insertive anal sex (1037/1512, 68.6%), had exclusively gay sexual partners (2394/3455, 69.3%), had more than 2 sexual partners in the past 3 months (1001/1419, 70.5%), frequently used rush poppers during sex (529/698, 75.8%), were consistently aware of their sexual partner’s HIV status (1371/2007, 68.3%), had a history of STIs (96/126, 76.2%), had HIV testing through mail reagent self-testing (1592/2169, 73.4%), tested HIV-positive (52/66, 78.8%), and had been previously tested for HIV (592/865, 68.4%) (Table 1).

**Table 1:** Social demographic and behavioral characteristics among men who have sex with men who were recruited through mobile social media promotion and volunteer-driven recruitment strategy

	Variables	Participants, n (%)		Number of mobile recruitments, n (%)		Number of volunteer-driven recruitment, n (%)		Chi-square (*df*)			*P* value		
Overall			4600 (100)		3035 (66.0)		1565 (34.0)						
**Age (years), IQR 27 (23-32)**										9.5 (2)			.008
	<20	306 (6.7)		190 (62.1)		116 (37.9)							
	20-29	2719 (59.1)		1842 (67.7)		877 (32.3)							
	30 years or older	1575 (34.2)		1003 (63.7)		572 (36.3)							
**Marital status**										3.5 (2)			.172
	Single	3606 (78.4)		2404 (66.7)		1202 (33.3)							
	Married	857 (18.6)		544 (63.5)		313 (36.5)							
	Divorced or separated	137 (3.0)		87 (63.5)		50 (36.5)							
**Education** **level**										1.2 (3)			.750
	High school and below	1175 (25.5)		760 (64.7)		415 (35.3)							
	College	1175 (25.5)		779 (66.3)		396 (33.7)							
	Bachelor’s degree	1826 (39.7)		1213 (66.4)		613 (33.6)							
	Master's degree or above	424 (9.2)		283 (66.7)		141 (33.3)							
**Occupation**										15.9 (3)			.001
	Company employee	1605 (34.9)		1111 (69.2)		494 (30.8)							
	Student	836 (18.2)		555 (66.4)		281 (33.6)							
	Freelance	612 (13.3)		374 (61.1)		238 (38.9)							
	Others	1547 (33.6)		995 (64.3)		552 (35.7)							
**Sex roles**										11.3 (2)			.004
	Receptive anal sex	1003 (21.8)		675 (67.3)		328 (32.7)							
	Both	2085 (45.3)		1323 (63.5)		762 (36.5)							
	Insertive anal sex	1512 (32.9)		1037 (68.6)		475 (31.4)							
**Sexuality of partner**										67.9 (1)			<.001
	Gay	3455 (75.1)		2394 (69.3)		1061 (30.7)							
	Bisexual	1145 (24.9)		641 (56.0)		504 (44.0)							
**Number of sexual partners in the past 3 months**													
	0	903 (19.6)		463 (51.3)		440 (48.7)		109.2 (2)			<.001		
	1	2278 (49.5)		1571 (69.0)		707 (31.0)							
	2	1419 (30.8)		1001 (70.5)		418 (29.5)							
**Condom use in the past 3 months**										75.3 (3)			<.001
	Every time	2364 (51.4)		1606 (67.9)		758 (32.1)							
	Sometimes use	1122 (24.4)		777 (69.3)		345 (30.7)							
	No sex	822 (17.9)		438 (53.3)		384 (46.7)							
	Never use	292 (6.3)		214 (73.3)		78 (26.7)							
**Rush poppers** **usage frequency during sex activity**										46.2 (2)			<.001
	Frequently use	698 (15.2)		529 (75.8)		169 (24.2)							
	Occasional use	712 (15.5)		495 (69.5)		217 (30.5)							
	Never	3190 (69.3)		2011 (63.0)		1179 (37.0)							
**Awareness of partner’s HIV status**										36.8 (2)			<.001
	Yes	2007 (43.6)		1371 (68.3)		636 (31.7)							
	Part	1516 (33.0)		1036 (68.3)		480 (31.7)							
	No	1077 (23.4)		628 (58.3)		449 (41.7)							
**History of STIs^a^**										6.0 (1)			.014
	No	4474 (97.3)		2939 (65.7)		1535 (34.3)							
	Yes	126 (2.7)		96 (76.2)		30 (23.8)							
**Awareness of PrEP^b^**										108.9 (1)			<.001
	Yes	3298 (71.7)		2327 (70.6)		971 (29.4)							
	No	1302 (28.3)		708 (54.4)		594 (45.6)							
**Awareness of PEP^c^**										118.4 (1)			<.001
	Yes	3402 (74.0)		2398 (70.5)		1004 (29.5)							
	No	1198 (26.0)		637 (53.2)		561 (46.8)							
**HIV testing pathway**										100.7 (1)			<.001
	Mailed self-testing	2169 (47.2)		1592 (73.4)		577 (26.6)							
	Offline testing	2431 (52.8)		1443 (59.4)		988 (40.6)							
**HIV status**										4.9 (1)			.027
	Positive	66 (1.4)		52 (78.8)		14 (21.2)							
	Negative	4534 (98.6)		2983 (65.8)		1551 (34.2)							
**Ever first-time HIV testing**										2.9 (1)			.090
	No	3735 (81.2)		2443 (65.4)		1292 (34.6)							
	Yes	865 (18.8)		592 (68.4)		273 (31.6)							

^a^STIs: sexually transmitted infections.

^b^PrEP: pre-exposure prophylaxis.

^c^PEP: postexposure prophylaxis.

Overall, among the MSM recruited through social media, 78.9% (2394/3035) had gay sexual partners, 33% (1001/3035) had more than 2 sexual partners, 17.4% (529/3035) frequently used rush poppers during sex, 3.2% (96/3035) had a history of STIs, 76.7% (2327/3035) had awareness of PrEP, 79% (2398/3035) had awareness of PEP, 1.7% (52/3035) tested HIV-positive, and 19.5% (592/3035) had been tested for HIV for the first time, all of which were higher than those of the volunteer recruitment (Table 2).

**Table 2:** Factors associated with mobile social media promotion compared with volunteer-driven recruitment strategy among men who have sex with men.

Variables			Number of mobile social media recruitments, n (%)		Number of volunteer-driven recruitments, n (%)		aOR^a^ (95% CI)		*P* value
Overall			3035 (100)		1565 (100)				
**Sexuality of partner**									
	Gay	2394 (78.9)		1061 (67.8)		1.23 (1.05-1.45)		.013	
	Bisexual	641 (21.1)		504 (32.2)		1			
**Number of sexual partners in the past 3 months**									
	0	463 (15.3)		440 (28.1)		1			
	1	1571 (51.8)		707 (45.2)		1.66 (1.38-1.00)		<.001	
	2	1001 (33.0)		418 (26.7)		1.74 (1.42-2.11)		<.001	
**Rush poppers** **usage frequency during sex activity**									
	Frequently use	529 (17.4)		169 (10.8)		1.39 (1.14-1.99)		.001	
	Occasional use	495 (16.3)		217 (13.9)		1.12 (0.93-1.35)		.225	
	Never	2011 (66.3)		1179 (75.3)		1			
**History of STIs^b^**									
	No	2939 (96.8)		1535 (98.1)		1			
	Yes	96 (3.2)		96 (1.9)		1.56 (1.02-2.39)		.040	
**Awareness of PrEP^c^**									
	Yes	2327 (76.7)		971 (62.0)		1.42 (1.19-1.71)		<.001	
	No	708 (23.3)		594 (38.0)		1			
**Awareness of PEP^d^**									
	Yes	2398 (79.0)		1004 (64.2)		1.49 (1.24-1.79)		<.001	
	No	637 (21.0)		561 (35.8)		1			
**HIV status**									
	Positive	52 (1.7)		14 (0.9)		2.02 (1.10-3.72)		.024	
	Negative	2983 (98.3)		1551 (99.1)		1			
**Ever first-time HIV testing**									
	No	2443 (80.5)		1292 (82.6)		1			
	Yes	592 (19.5)		273 (17.4)		1.28 (1.09-1.52)		.003	

^a^aOR: adjusted odds ratio.

^b^STI: sexually transmitted infections.

^c^PrEP: pre-exposure prophylaxis.

^d^PEP: postexposure prophylaxis.

In multivariate logistic regression analysis, the following factors were significantly associated with mobile social media recruitment: having only gay sexual partners (aOR 1.23, 95% CI 1.05-1.45), having more than 2 sexual partners in the past 3 months (aOR 1.74, 95% CI 1.42-2.11), frequently using rush poppers during sex (aOR 1.39, 95% CI 1.14-1.99), having a history of STIs (aOR 1.56, 95% CI 1.02-2.39), having awareness of PrEP (aOR 1.42, 95% CI 1.19-1.71), having awareness of PEP (aOR 1.49, 95% CI 1.24-1.79), testing positive for HIV (aOR 2.02, 95% CI 1.10-3.72), and undergoing HIV testing for the first time (aOR 1.28, 95% CI 1.09-1.52) (Table 2). MSM who were recruited by the mobile platform were more likely (aOR 2.02, 95% CI 1.77-2.31) to take the mailed HIV self-test (Table 3).

**Table 3:** Factors associated with mailed self-testing compared with offline testing among men who have sex with men.

Variables		Participants, n (%)	Number of mailed self-testing, n (%)	Number of offline testings, n (%)	Chi-square (*df*)	*P* value	aOR^a^ (95% CI)
Overall		4600 (100)	2169 (47.2)	2431 (52.8)			
**Age (years), IQR 27 (23-32)**					97.7 (2)	<.001	
	<20	306 (6.7)	132 (43.1)	174 (56.9)		.092	1.30 (0.96-1.76)
	20-29	2719 (59.1)	1444 (53.1)	1275 (46.9)		<.001	1.43 (1.22-1.68)
	≥30	1575 (34.2)	593 (37.7)	982 (62.3)			1
**Marital status**					99.8 (2)	<.001	
	Single	3606 (78.4)	1836 (50.9)	1770 (49.1)			1
	Married	857 (18.6)	275 (32.1)	582 (67.9)		<.001	0.59 (0.49-0.72)
	Divorced or separated	137 (3.0)	58 (42.3)	79 (57.7)		.876	1.03 (0.71-1.50)
**Education level**					122.8 (3)	<.001	
	High school and below	1175 (25.5)	402 (34.2)	773 (65.8)			1
	College	1175 (25.5)	550 (46.8)	625 (53.2)		<.002	1.34 (1.11-1.61)
	Bachelor’s degree	1826 (39.7)	993 (54.4)	833 (45.6)		<.001	1.62 (1.35-1.93)
	Master’s degree or above	424 (9.2)	224 (52.8)	200 (47.2)		<.001	1.67 (1.29-2.15)
**Occupation**					59.8 (3)	<.001	
	Company employee	1605 (34.9)	865 (53.9)	740 (46.1)		.006	1.25 (1.07-1.46)
	Student	836 (18.2)	411 (49.2)	425 (50.8)		.685	0.96 (0.78-1.18)
	Freelance	612 (13.3)	250 (40.8)	362 (59.2)		.485	0.93 (0.76-1.14)
	Others	1547 (33.6)	643 (41.6)	904 (58.4)			1
**Sex roles**					29.5 (2)	<.001	
	Receptive anal sex	1003 (21.8)	528 (52.6)	475 (47.4)			1
	Both	2085 (45.3)	896 (43.0)	1189 (57.0)		<.001	0.64 (0.54-0.76)
	Insertive anal sex	1512 (32.9)	745 (49.3)	767 (50.7)		.258	0.91 (0.76-1.08)
**Sexuality of partner**					0.2 (1)	.638	
	Gay	3455 (75.1)	1636 (47.4)	1819 (52.6)			1
	Bisexual	1145 (24.9)	533 (46.6)	612 (53.4)		<.001	1.45 (1.22-1.72)
**Number of sexual partners in the past 3 months**					24.3 (2)	<.001	
	0	903 (19.6)	402 (44.5)	501 (55.5)			1
	1	2278 (49.5)	1021 (44.8)	1257 (55.2)		.822	1.03 (0.82-1.29)
	2	1419 (30.8)	746 (52.6)	673 (47.4)		.013	1.34 (1.05-1.72)
**Condom use in the past 3 months**					24.6 (3)	<.001	
	Every time	2364 (51.4)	1050 (44.4)	1314 (55.6)			1
	Sometimes use	1122 (24.4)	594 (52.9)	528 (47.1)		<.001	1.49 (1.28-1.74)
	No sex	822 (17.9)	376 (45.7)	446 (54.3)		.095	1.22 (0.97-1.53)
	Never use	292 (6.3)	149 (51.0)	143 (49.0)		.013	1.39 (1.07-1.80)
**Rush poppers** **usage frequency during sex activity**					36.3 (2)	<.001	
	Frequently use	698 (15.2)	278 (39.8)	420 (60.2)			1
	Occasional use	712 (15.5)	397 (55.8)	315 (44.2)		<.001	1.93 (1.54-2.41)
	Never	3190 (69.3)	1494 (46.8)	1696 (53.2)		<.001	1.57 (1.31-1.88)
**Awareness of partner’s HIV status**					8.5 (2)	.014	
	Yes	2007 (43.6)	956 (47.6)	1051 (52.4)			^—e^
	Part	1516 (33.0)	745 (49.1)	771 (50.9)			—
	No	1077 (23.4)	468 (43.5)	609 (56.5)			—
**History of STIs^b^**					6.8 (1)	.009	
	No	4474 (97.3)	2124 (47.5)	2350 (52.5)		<.003	1.80 (1.22-2.65)
	Yes	126 (2.7)	45 (35.7)	81 (64.3)			1
**Awareness of PrEP^c^**					3.6 (1)	.058	
	Yes	3298 (71.7)	1584 (48.0)	1714 (52.0)			—
	No	1302 (28.3)	585 (44.9)	717 (55.1)			—
**Awareness of PEP^d^**					5.8 (1)	.016	
	Yes	3402 (74.0)	1640 (48.2)	1762 (51.8)			—
	No	1198 (26.0)	529 (44.2)	669 (55.8)			—
**HIV status**					25.0 (1)	<.001	
	Positive	66 (1.4)	11 (16.7)	55 (83.3)			1
	Negative	4534 (98.6)	2158 (47.6)	2376 (52.4)		<.001	5.04 (2.55-9.96)
**Ever first-time HIV testing**					51.4 (1)	<.001	
	No	3735 (81.2)	1856 (49.7)	1879 (50.3)		<.001	1.75 (1.48-2.06)
	Yes	865 (18.8)	313 (36.2)	552 (63.8)			1
**Recruitment**					100.7 (1)	<.001	
	Volunteer-driven	1565 (34.0)	577 (36.9)	988 (63.1)			1
	Mobile recruitment	3035 (66.0)	1592 (52.5)	1443 (47.5)		<.001	2.02 (1.77-2.31)

^a^aOR: adjusted odds ratio.

^b^STI: sexually transmitted infections.

^c^PrEP: pre-exposure prophylaxis.

^d^PEP: postexposure prophylaxis.

^e^Not included in multivariate logistic regression analysis.

## Discussion

### Principal Results

This study compared the sociodemographic and behavioral characteristics of MSM who were recruited through 2 different recruitment strategies by community organizations to engage in HIV testing in Zhejiang Province, China. The strategies examined were a mobile social media promotion strategy and a volunteer-driven recruitment strategy. The findings indicated that 66% (3035/4600) of MSM were recruited through mobile social media promotion, while both strategies together reached 18.8% (865/4600) of MSM who had never been tested for HIV. Consistent with previous studies [18], community organizations proved effective in increasing HIV testing rates among MSM, particularly those who had not been tested before. The implementation of both strategies successfully expanded intervention coverage and recruited MSM who had not been previously tested for HIV. Furthermore, ongoing support for community organizations is recommended to facilitate the implementation of MSM prevention and control initiatives.

### Comparison With Previous Work

Our findings revealed a higher proportion of individuals undergoing their first HIV test (592/3035, 19.5% vs 273/1565, 17.4%) and a higher prevalence of HIV-positive results (52/3035, 1.7% vs 14/1565, 0.9%) among those engaged via mobile social media promotions compared to those recruited through volunteer-driven initiatives. These observations are consistent with previous studies and empirical data. A meta-analysis demonstrated a 50% increase in HIV testing uptake following social media interventions [19], and another study showed a 22% increase in testing through mobile platforms [20], particularly among MSM engaging in high-risk behaviors. In these populations, the HIV-positive rate was 3.5% [21]. These findings suggest that mobile social media promotion may be an effective approach for recruiting MSM, improving HIV-testing coverage, and identifying individuals infected with HIV, particularly those who have not previously undergone HIV testing. As the proportion of MSM who use the internet and dating software continues to grow, it may be beneficial to expand the use of mobile social media promotion strategies to enhance HIV testing among MSM and facilitate the identification of more individuals infected with HIV. This could be a crucial step toward achieving the goal of diagnosing 95% of individuals infected with HIV by 2030.

Similar to previous studies [22,23], our findings indicate that MSM recruited through mobile social media were more likely to use mail-based HIV self-testing kits. Mobile promotion strategies can facilitate the recruitment of MSM who have not accessed HIV testing services by providing convenient mail-in self-testing kits, thus increasing overall testing and identifying more individuals infected with HIV. Another study demonstrated that HIV self-testing services have the potential to enhance test uptake and reduce undiagnosed infections [24]. It would be beneficial to provide targeted outreach to MSM, such as offering HIV self-testing [25]. The utilization of social media platforms may prove effective in enhancing HIV self-testing rates among MSM aged ≤35 years [26]. Nevertheless, research has indicated that mobile social media may not necessarily increase the rate of HIV testing [27]. This may be attributed to the fact that the testing rate may not be enhanced by repeated HIV publicity and intervention in large cities but rather in areas where HIV testing is not easily accessible and where supportive gay social networks and health professionals are scarce. Therefore, priority should be given to implementing mobile social media promotion in areas lacking LGBTQ+ organizations or HIV health services. This approach can facilitate MSM recruitment, thereby increasing intervention coverage and improving HIV testing. Concurrently, it is also recommended to continue increasing publicity about HIV self-testing and to provide convenient self-testing services.

Consistent with other studies [28,29], we found that MSM recruited via mobile social media promotion were more aware of PrEP and PEP. Dating apps and social media have been identified as underutilized tools for increasing PrEP awareness, uptake, and knowledge among MSM [28,29]. PrEP and PEP represent effective prevention tools for individuals at high risk of HIV exposure, effectively reducing HIV infection and transmission [30]. Since 2021, PrEP and PEP have been widely used in China [31], with much of the advertising and promotion happening mobile. This has substantially increased awareness among MSM using the Internet. This study demonstrated that most MSM initially learned about PrEP through mobile sources [32]. Efforts should be made to further promote PrEP and PEP through mobile platforms while also disseminating preventive information through offline volunteer-driven strategies.

Our findings also revealed that MSM recruited mobile were more likely to report using rush poppers, a recreational drug that has gained considerable popularity among MSM [33], and their rate of use has continued to increase in China [34]. The use of rush poppers has been linked to abnormal arousal and pleasure, and prolonged sexual activity; these factors may increase the likelihood of engaging in unprotected sexual activity with consequent adverse outcomes such as HIV infection [30] and transmission [35]. Furthermore, a greater proportion of MSM seeking sexual activity through social media have reported recreational drug use compared to those using traditional venues [21]. Therefore, it is recommended that internet-based HIV interventions include information on the risks associated with rush poppers and their link to HIV transmission to raise awareness and reduce high-risk behaviors.

The study also revealed that MSM recruited mobile had more sexual partners and a higher prevalence of STIs compared to those recruited through volunteer-driven strategies. A meta-analysis conducted in China revealed that MSM who engage in substance abuse are more likely to seek sexual partners through the internet or social media and engage in unprotected anal intercourse [36]. One potential explanation for this is that the volunteer-driven strategy for MSM tends to focus on offline recruitment in venues such as bars, parks, and bathhouses, where social circles are relatively limited. The advent of the Internet has provided MSM with more opportunities to search for sexual partners. Mobile social media have demonstrated efficacy as a tool for increasing PrEP use [29] and HIV-testing uptake [32]. While PrEP reduces the risk of HIV infection, it does not prevent STIs [37] and may even increase the risk due to reduced condom use and increased high-risk sexual behaviors [38]. Therefore, alongside PrEP promotion, other targeted interventions promoting condom use and awareness of STI risks should be integrated into internet-based HIV prevention efforts.

### Limitations

First, the participants recruited by the community organization were primarily younger MSM who use the internet, meaning older MSM (particularly those over 50) were underrepresented. Future research should explore recruitment strategies targeting older MSM who do not engage mobile. Second, the costs associated with the human and financial resources invested in these recruitment strategies were not assessed. Further research on the cost-effectiveness of these strategies is necessary. Finally, the study relied on routine surveillance questionnaires, which may not have captured all relevant factors influencing the effectiveness of the 2 investigated recruitment strategies.

### Conclusions

Community organizations effectively use mobile social media promotion and volunteer-driven strategies to recruit MSM, playing an important role in improving HIV testing and identifying undiagnosed individuals infected with HIV. There are notable differences in recruitment outcomes between the 2 strategies, with mobile social media promotion leading to a higher proportion of first-time HIV testing and HIV-positive individual identification. In areas where LGBTQ+ organizations or HIV health services are scarce, mobile social media promotion should be prioritized to increase MSM recruitment and expand the coverage of HIV interventions.

## Abbreviations

aOR: adjusted odds ratio
MSM: men who have sex with men
PrEP: pre-exposure prophylaxis
PEP: postexposure prophylaxis
STIs: sexually transmitted infections

## Data Availability

The datasets generated during and/or analyzed during this study are available from the corresponding author on reasonable request.
